# Investigation of Structural, Physical, and Attenuation Parameters of Glass: TeO_2_-Bi_2_O_3_-B_2_O_3_-TiO_2_-RE_2_O_3_ (RE: La, Ce, Sm, Er, and Yb), and Applications Thereof

**DOI:** 10.3390/ma15155393

**Published:** 2022-08-05

**Authors:** Nehal Elkhoshkhany, Samir Marzouk, Mohammed El-Sherbiny, Heba Ibrahim, Bozena Burtan-Gwizdala, Mohammed S. Alqahtani, Khalid I. Hussien, Manuela Reben, El Sayed Yousef

**Affiliations:** 1 Physics Department, College of Arts and Sciences at Tabrjal, Jouf University, Sakaka 72388, Saudi Arabia; 2 Department of Material Science, Institute of Graduate Studies and Researches, Alexandria University, 163 Horreya Avenue, Shatby, Alexandria 21526, Egypt; 3 Department of Basic and Applied Science, Faculty of Engineering and Technology, Arab Academy of Science and Technology, Cairo 11511, Egypt; 4 Institute of Physics, Cracow University of Technology, ul. Podchorazych 1, 30-084 Cracow, Poland; 5 Department of Radiological Sciences, College of Applied Medical Sciences, King Khalid University, Abha 61421, Saudi Arabia; 6 BioImaging Unit, Space Research Centre, Department of Physics and Astronomy, University of Leicester, Leicester LE1 7RH, UK; 7 Department of Medical Physics and Instrumentation, National Cancer Institute, University of Gezira, Wad Medani 2667, Sudan; 8 Faculty of Materials Science and Ceramics, AGH—University of Science and Technology, al. Mickiewicza 30, 30-059 Cracow, Poland; 9 Physics Department, Faculty of Science, King Khalid University, Abha 61413, Saudi Arabia; 10 Research Center for Advanced Materials Science (RCAMS), King Khalid University, Abha 61413, Saudi Arabia

**Keywords:** oxide glass, rare earth, optical energy gap, physical parameter, FTIR, attenuation

## Abstract

A novel series of glass, consisting of B_2_O_3_, Bi_2_O_3_, TeO_2_, and TiO_2_ (BBTT) containing rare earth oxide RE_2_O_3_, where RE is La, Ce, Sm, Er, and Yb, was prepared. We investigated the structural, optical, and gamma attenuation properties of the resultant glass. The optical energy bands, the linear refractive indices, the molar refractions, the metallization criteria, and the optical basicity were all determined for the prepared glass. Furthermore, physical parameters such as the density, the molar volume, the oxygen molar volume, and the oxygen packing density of the prepared glass, were computed. Both the values of density and optical energy of the prepared glass increased in the order of La_2_O_3_, Ce_2_O_3_, Sm_2_O_3_, Er_2_O_3_, and then Yb_2_O_3_. In addition, the glass doped with Yb_2_O_3_ had the lowest refractive index, electronic polarizability, and optical basicity values compared with the other prepared glass. The structures of the prepared glass were investigated by the deconvolution of infrared spectroscopy, which determined that TeO_4_, TeO_3_, BO_4_, BO_3_, BiO_6_, and TiO_4_ units had formed. Furthermore, the structural changes in glass are related to the ratio of the intensity of TeO_4_/TeO_3_, depending on the type of rare earth. It is also clarified that the resultant glass samples are good attenuators against low-energy radiation, especially those that modified by Yb_2_O_3_, which exhibited superior shielding efficiency at energies of 622, 1170, and 1330 keV. The optical and gamma ray spectroscopy results of the prepared glass show that it is a good candidate for nonlinear optical fibers, laser solid material, and optical shielding protection.

## 1. Introduction

Rare earth (RE) elements, such as La, Ce, Pr, Nd, Sm, Eu, Gd, Tb, Dy, Ho, Er, Tm, and Yb have many multilateral applications in advanced technology [1,2]. Recently, glass doped with rare earth (RE) ions has attracted interest due to its utility in various applications in the development of opto-electronic devices such as planar waveguides, fluorescent display devices, optical fibers, visible lasers, optical detectors, and optical amplifiers [3]. Tellurite glass, especially, has good optical characteristics such as a high dielectric constant, high refractive index, wide optical transmission windows, good semiconducting properties [4], low melting point, and low phonon energy ($\sim 700\;{\mathrm{cm}}^{-1})$, as compared to borate glass which has high phonon energy and exists at a range of 1300–1500 ${\mathrm{cm}}^{-1}$ [4,5,6]. Glass based on TeO_2_ has a weak Te–O bond as compared to other composite glass; the former can easily be broken, which is useful for accommodating metal oxides and rare earth ions [7]. Combined B_2_O_3_ and TeO_2_ (borotellurite) glass exhibits high thermal stability, low phonon energy, easy fabrication, and chemical durability [8]. Borotellurite glass is widely used in several applications, particularly in opto-acoustics, radiation shielding, and micro-electronics [8]. Glass that contains heavy metal oxides (HMO), such as Bi_2_O_3,_ has high nonlinear second/third-harmonic generation, exhibits high thermal expansion, high density, and IR transmission [8,9,10]. TiO_2_ incorporated into the glass matrix improves covalent bond formation and reinforces a continuous network consisting of TiO_6_ with an increase in the amount of bridging oxygen (BO) [10,11,12,13]. Moreover, TiO_2_ improves the properties of chemical resistance and thermal stability when added to tellurite glass. Nupur Gupta et al. [10] reported that the addition of both TiO_2_ and Bi_2_O_3_ to borotellurite glass leads to a decrease in the optical energy gap due to the formation of nonbridging oxygen (NBO), and the glass transition temperature, T_g_, was increased. The addition of La_2_O_3_ to tellurite glass enhances the stability of the glass against crystallization. Cerium-ion-doped glass holds several applications in biosensors, solid oxide fuel cells, dielectric materials, and blue luminescent optical systems [14]. Depending on the excitation wavelength, Sm_2_O_3_-doped glass displays and emits a strong orange-red luminescence in the visible range [15], associated with ^4^G_5/2_, ^6^H_9/2_ transition. Er_2_O_3_-doped glass is considered to be a candidate for use in the manufacturing of optical amplifiers (EDFA_S_) and in the field of optical communications [16]. Furthermore, several researchers [17,18,19,20,21] have investigated tellurium-based glass for possible uses in nuclear-radiation-shielding applications. The shielding effectiveness for tellurium-based glass has been studied, with the results showing that glass containing 60 Mol % of lead oxide and heavy oxide, and recording the highest density resulted in higher linear attenuation values and superior shielding material protection with perfect shielding efficiency. In the same trend, our research group [22] developed a computation tool (MIKE) for estimating and analyzing the shielding and optical parameters for different types of shielding materials. Therefore, the purpose of the present research was to study the physical parameters as well as the optical and attenuation properties of novel tellurite glass structures modified with various rare earths ions: La^3+^, Ce^3+^, Sm^3+^, Er^3+^, and Yb^3+^.

## 2. Experimental Section

**Figure 1:**
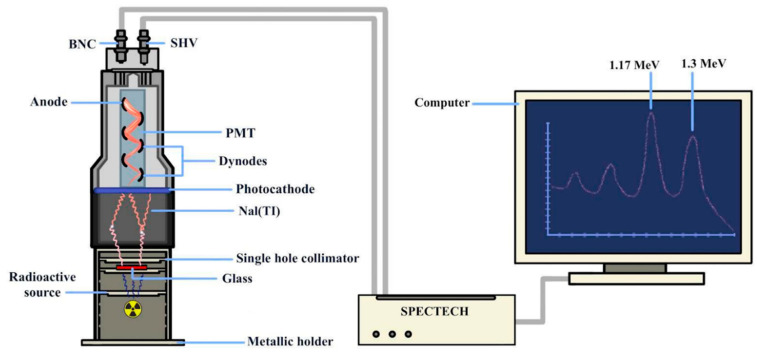
Experimental setup used for measuring the shielding parameters of the prepared samples.

The prepared glass (BBTTER) with a composition of 25B_2_O_3_–20Bi_2_O_3_–45TeO_2_–7TiO_2_ (BBTT), modified by 3RE_2_O_3_ in mol%, where RE is La; Ce; Sm; Er; or Yb, was manufactured using the melt-quenching method. Chemical powders of TeO_2_, B_2_O_3_, Bi_2_O_3_, TiO_2_, and RE_2_O_3_ (La_2_O_3_, Ce_2_O_3_, Sm_2_O_3_, Er_2_O_3_, and Yb_2_O_3_) were homogenized and melted at 930 °C for 30 min in an electric furnace. We used a platinum crucible while melting to obtain a homogeneous mixture, stirring the mixture several times. Then, each melt was put into a polished stainless-steel container and annealed at 300 °C. The prepared glass samples are coded as: BBTTLa, BBTTCe, BBTTSm, BBTTEr, and BBTTYb, as shown in Table 1. Al_2_O_3_ powder of 600 grade was used to polish the glass samples. The value density of the samples was determined according to Archimedes’ method. The powder X-ray diffraction pattern (XRD) was determined using a Philips PW (1140) diffractometer, and a copper target (Kα = 1.54 Å) was used to study the amorphous properties of prepared glass. A double-beam UV–Visible spectrophotometer (JASCO Corp, v-570, Rel-00, Tokyo, Japan) was used to determine the optical absorption spectrum of the glass. The refractive index was determined with a prism coupler (Metricon Model 2010, Pennington, NJ, USA). Structural characterization of the glass was carried out using FTIR absorbance spectra (Perkin-Elmer spectrometer). For the prepared glass, the half-value layer, both the linear and mass attenuation coefficients, as well as the mean free pass were measured using a NaI detector system (SPECTECH-NaI 1.5 PX 1.5/2.0 IV, S/N 010723-6), with various gamma sources (Am^241^-5µCI-59.5 keV, Cs^137^-5µCI- 662 keV, Co^60^-5µCI-1170, and 1330 keV), which was connected to a computer and based on the multichannel analyzer. Figure 1 shows a collimated beam which was produced at the detector level using a variety of gamma sources (Am^241^-5µCI-59.5 keV, Cs^137^-5µCI-662 keV, Co^60^-5µCI-1170, and 1330 keV), according to the technique described in [4].

## 3. Results and Discussion

### 3.1. XRD, Physical Parameters, and UV–VIS–NIR Spectra

**Figure 2:**
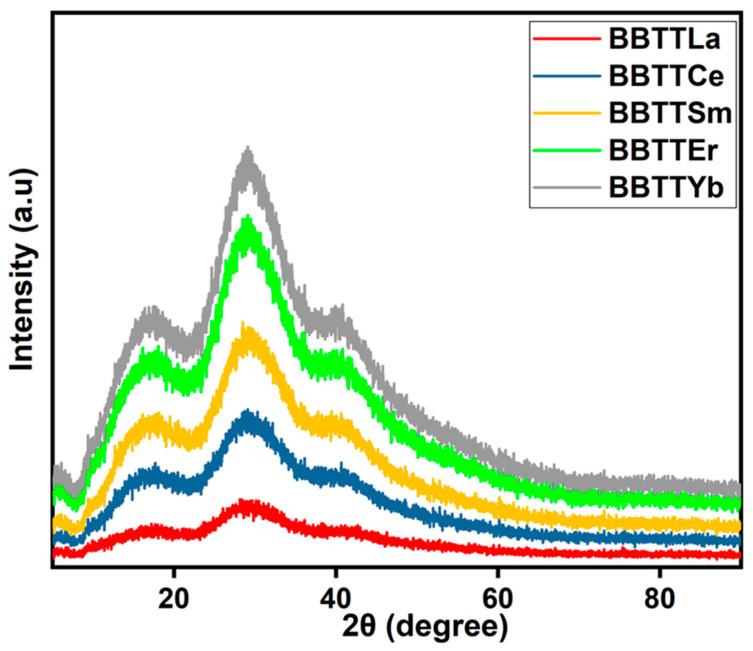
XRD profile of prepared glass.

The X-ray diffraction (XRD) patterns of the glass samples were measured to investigate the nature of the glass samples, as shown in Figure 2. The absence of any discrete or sharp diffraction peaks in these profiles and the existence of broad bands proves that all the prepared glass samples had an amorphous nature. The density value (ρ) was calculated using Equation (1) [23].

(1) $$\rho =\left(\frac{W_{a}}{W_{a}-W_{t}}\right)\cdot \rho_{t}\;(\mathrm{gm}/{\mathrm{cm}}^{3})$$

The weight of the glass sample in air is “W_a_”, whereas the weight of the glass immersed in reference liquid toluene is “W_t_”, where $\rho_{t}=0.864\;g\cdot {\mathrm{cm}}^{-3}$. The calculated density value of the prepared glass increased from 5.67 g·cm^−3^ to 6.32 g·cm^−3^, which corresponded to BBTTLa and BBTTYb glass, respectively. The results are shown in Table 1.

The molecular weights, M_w__t_, of rare earth compounds are ordered as follows: La_2_O_3_ < Ce_2_O_3_ < Sm_2_O_3_ < Er_2_O < Yb_2_O_3_, corresponding to 325.5, 328.24, 348.72, 382.52, and 394.08 g·mol^−1^, respectively. Thus, the density increased in the same trend, which means that the highest value of density occurred with Yb_2_O_3_ incorporated into the glass matrix; here, the network of the glass is more compact. It is possible to determine the glass sample’s molecular volume (V_m_) and its oxygen molar volume (V_o_) using Equations (2) and (3), respectively. These strongly depend on the value of the densities of the glass samples, tabulated in Table 2.

(2) $$V_{m}=\frac{\sum x_{i}{\;M}_{\mathrm{wti}}}{\rho }$$

where “M_wti_” is the molecular weight and “x_i_” is the fraction ratio of each oxide. The oxygen molar volume, V_O_, can be estimated by the relationship as follow:

(3) $$\;V_{O}=\left(\sum x_{i}{\;M}_{\mathrm{wti}}/\rho \right)(1/\left(\sum x_{i}{\;n}_{i}\right)$$

where “n_i_” is the number of oxygen atoms in each oxide [24]. The number of bonds per unit volume, n_b_, of the prepared glass and the average force constant ($\bar{F}$), calculated from Equations (4) and (5), respectively [25,26].

(4) $$\;n_{b}=\frac{N_{a}}{V_{m}\;}\sum x_{i}{\;n}_{f}$$

where n_f_ is the cation coordination number and N_a_ is the Avogadro’s number.

(5) $$\bar{F}=\frac{\sum x_{i}{\;n}_{f}{\;f}_{i}\;}{\sum x_{i}{\;n}_{f}}$$

The stretching force, f_i_, of the oxide, i, can be measured using the following formula:

(6) $$\;f_{i}=\frac{1.7}{r^{3}}$$

The molar volume, V_m_, decreased from 34.9 cm^3^/mol to 31.6 cm^3^/mol, which corresponded to BBTTLa and BBTTYb, respectively, and the glass structures became more compact due to a reduction in interatomic space or bond length between atoms (r_i_). The value of V_0_ decreased from 14.1 to 12.7 cm^3^·mol^−1^ by increasing both values of n_b_ from 6.5 to 7.14 × 10^22^ m^−3^ and $\bar{F}$ from 301.21 to 303.04 Nm^−1^, when replacing the modified rare earth oxide La_2_O_3_ by Yb_2_O_3_. Furthermore, the decrease in V_o_ may indicate a decrement in the formation of NBO atoms reported here. The values of V_m_, V_0_, n_b_, and $\bar{F}$ of the prepared glass are shown in Table 2. Equation (7) was used to compute the oxygen packing density, OPD, [23]:

(7) $$\mathrm{OPD}=1000\cdot C\cdot \left(\frac{\rho }{M}\right)$$

where “C” is the number of oxygen atoms per formula unit. The increase in the value of OPD from 71.06258 mol/liter to 78.40199 mol/liter of BBTTLa and BBTTYb, respectively, was associated with increases in the n_b_, $\bar{\;F}$, and M_wt_ values of rare earth oxides.

**Figure 3:**
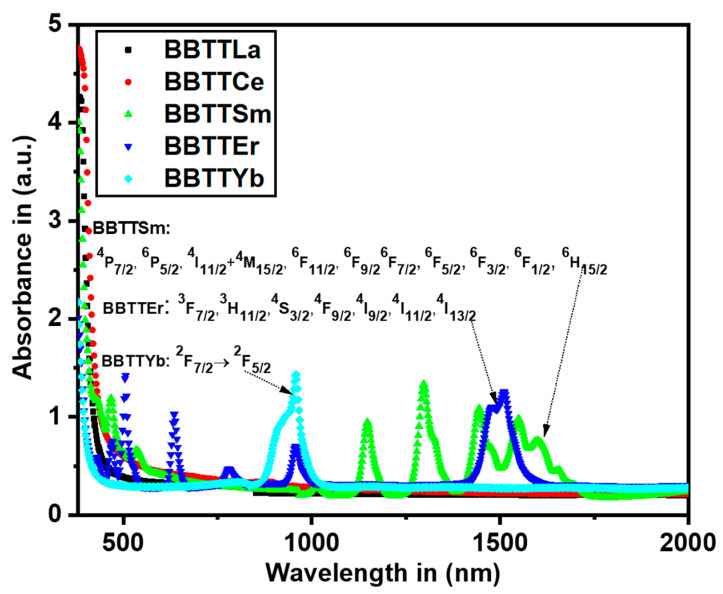
Absorbance spectra of prepared glass.

The optical absorption spectra of BBTTRE glass are shown in Figure 3. The results on the absorption edge provide important information on the transitions of the band structure of amorphous materials [27]. The absorption bands of BBTTEr glass were detected at 1512, 973, 800, 650, 550, 520, and 490 nm, which corresponded to the transitions from ^4^*I*_15/2_ to ^4^*I*_13/2_, ^4^*I*_11/2_, ^4^*I*_9/2_, ^4^*F*_9/2_, ^4^*S*_3/2_, ^2^*H*_11/2_, and ^4^*F*_7/2_, respectively.

The absorbance spectra of BBTTSm glass exhibited bands at 1620, 1551, 1485, 1379, 1230, 1085, 950, 476, 404, and 368 nm, attributed to the absorption ground state ^6^H_5/2_ to the excited states, ^6^*H*_15/2_, ^6^*F*_1/2_, ^6^*F*_3/2_, ^6^*F*_5/2_, ^6^*F*_7/2_, ^6^*F*_9/2_, ^6^*F*_11/2_, ^4^*I*_13/2_^+ 4^*M*_15/2_, ^4^*P*_5/2_, and ^4^*P*_7/2_, respectively. In addition, there was a strong absorption band transition of the level ^2^*F*_7/2_ to ^2^*F*_5/2_ of the Yb^3+^-ion-modified BBTTYb glass. The absorption coefficient, α(ν), of the fabricated glass was calculated using the absorbance spectra and the relationship shown below [28]:

(8) $$\alpha \left(\nu \right)=2.303\cdot A\cdot {\;d}^{-1}$$

where A represents the absorbance and d is the glass sample’s thickness in cm.

**Figure 4:**
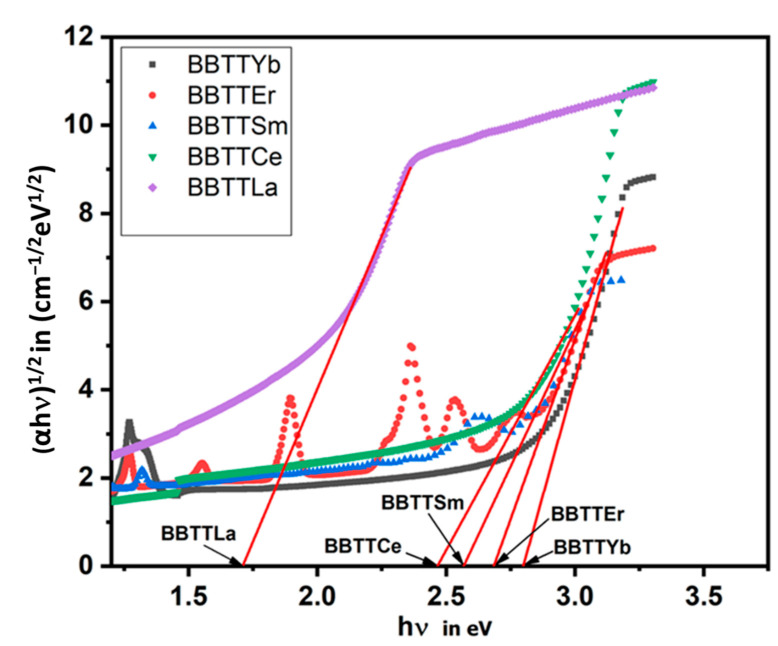
Relationship between (αhν)^1/2^ and hν of the prepared glass.

In amorphous material, optical transitions that occur at the absorption edge can be divided into two mechanisms: firstly, direct transitions are where the momentum of the electron from the valance to conduction band is preserved; secondly, there are indirect transitions where it is necessary to cooperate with the absorb/release phonon [29]. Mott and Davis [29] suggested a relationship between photon energy (hν) and absorption coefficient (α) to determine the indirect optical band gap, E_opt_, as shown in Equation (9).

(9) $$\left(\alpha \cdot h\nu \right)=B\;{\left(h\nu -\mathrm{Eopt}\right)}^{r}$$

where B is a constant known as the band tailing parameter, r, which depends on the type of mechanism transition (r = 2) associated with the allowed indirect transitions [30]. A graph was plotted for ${\left(\alpha h\nu \right)}^{1/2}$ versus (hν) to determine the indirect optical band gap, E_opt_, as shown in Figure 4. The calculated values of the indirect optical band gap, $E_{\mathrm{opt}}$, can be obtained by the extrapolation of the linear range of the curve with a linear axis at (Y-axis = 0), which represents the photon energy (hν) [31] and the value of E_opt_ for the prepared glass, as evaluated in Table 3. In the BBTTRE glass system, the formation of TeO_4_ caused oxygen anions to be tightly bound to the host materials; thus, the E_opt_ increased with a decrease in the number of NBO [31]. The value of E_opt_ depends on the structure of the prepared glass TBBT modified by 3RE_2_O_3_ in mol%, where RE is La, Ce, Sm, Er, or Yb. From the results presented in Table 3, the values of E_opt_ increase from 2.1 to 2.81 eV. The E_opt_ value increased as a result of more bridging oxygen being present (BO) and the decrease in the number of NBO, as confirmed in the FTIR results of the prepared glass discussed here. The value of the refractive index, n, molar polarizability, $\alpha_{m}$, molar refraction, $R_{m}$, oxide ion polarizability, ($\alpha_{o}^{-2}$), and the value of optical basicity, (Λ), are important parameters for the fabrication of optical devices, especially fiber optic and laser material. Therefore, we determine these parameters of the studied glass by using the subsequent equations [32]:

(10) $${\;\alpha }_{m}=\left(\frac{3}{4{\pi N}_{A}}\right)R_{m}$$

(11) $${\;R}_{m}=\left(\frac{n^{2}-1}{n^{2}+2}\right)V_{m}$$

(12) $$\alpha_{o}^{-2}=\left[\frac{V_{m}}{2.52}\left(1-\sqrt{\frac{\mathrm{Eopt}}{20}}\right)-\sum {p\alpha }_{i}\right]q^{-1}$$

(13) $$\Lambda =1.67\left[1-\frac{1}{\alpha_{o}^{-2}}\right]$$

where $N_{A}$ is Avogadro’s number, p is the cation number, and q denotes the number of ions of oxygen. The values of n, R_m_,${\;\alpha }_{m}$, and Λ depend on the polarizability of ions; the type of RE was La, Ce, Sm, Er, or Yb, and the prepared BBTT glass was modified with 3RE_2_O_3_ in mol%. The values of n, R_m_,${\;\alpha }_{m}$, and Λ decreased when the host glass network BBTT was modified with regard to the free ion polarizability due to internal contact and the polarizability of oxide rare earth ions decreased. High oxide ion polarizability and optical basicity are also closely related to the superior optical characteristics of tellurite glass. Herein, rare earth oxides had an order of polarizability of cation, α_i_, of (La_2_O_3_ = 1.32 Ă^3^), (Ce_2_O_3_ = 1.28 Ă^3^), (Sm_2_O_3_ = 1.16 Ă^3^), (Er_2_O_3_ = 0.89 Ă^3^), and (Yb_2_O_3_ = 0.86 Ă^3^) [33]. Furthermore, the values of optical basicity, Λ, in TeO_2_-based glass, i.e., Λ_TeO4_^0^ = 0.99, Λ_TeO4_^−^ = 1.23, and Λ_TeO3_^−^ = 0.82, were estimated by Dimitrov and Komatsu [33]. This demonstrated the polarizability of the TeO_3_ unit to be substantially lower that of the TeO_4_ unit, supporting the order of Λ_TeO4_^−^ > Λ_TeO4_^0^ > Λ_TeO3_^−^. Hence, highly distorted TeO_4_ tbp units with NBOs and different Te–O bond lengths should produce significant electrical polarizabilities. It is therefore of interest to clarify the fraction of TeO_4_^−^ units created in the order La_2_O_3_ > Ce_2_O_3_ > Sm_2_O_3_ > Er_2_O_3_ > Yb_2_O_3_ for modifying the BBTT glass matrix.

The metallization criterion, M, for BBTTRE glass was estimated as follow:

(14) $$M=1-\frac{R_{m}}{V_{m}}$$

The change in rare earth La_2_O_3_ → Ce_2_O_3_ → Sm_2_O_3_ → Er_2_O_3_ → Yb_2_O_3_ → La_2_O_3_ modified host matrix TBBT, causing a decrease in the width of the valance band and an increase in M, and consequently, an increase in the optical energy band gap. As shown in Table 3, the BBTTLa glass had the largest value of M and the smallest value of E_opt_. In contrast, the BBTTYb glass had the smallest value of M and the highest value of E_op__t_.

### 3.2. Structural Categorization of Glass Using FTIR Spectra

**Figure 5:**
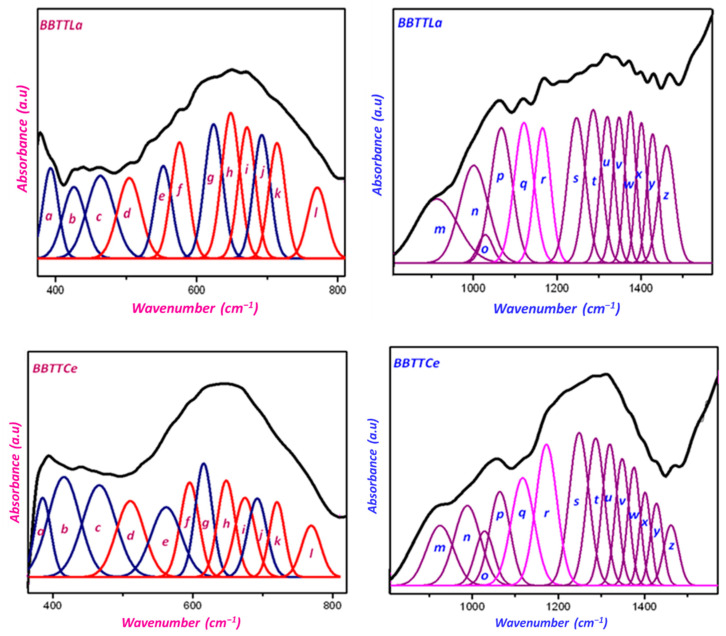

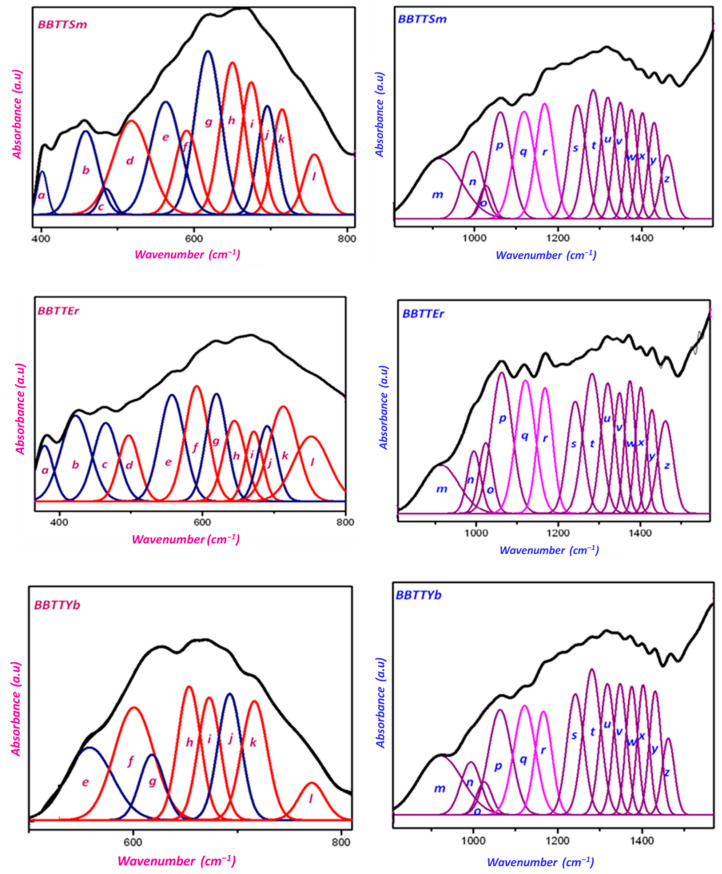
Deconvoluted FTIR spectra of the prepared glass.

The FTIR spectra of the investigated glass were measured; consequently, these were deconvoluted using Gaussian fitting into several Gaussian peaks marked as (a − x) bands, as shown Figure 5. Bands of 370–400 cm^−1^ have been linked to the stretching vibration mode of Bi–O–Bi linkages [34]. The bands observed at around 430, 440, 458, 425, and 430 cm^−1^ are attributable to La–O, Ce–O, Sm–O, Er–O, and Yb–O stretching vibrations, respectively [35,36]. Clearly visible peaks in the range of 463–480 cm^−1^ can be attributed to the bridging anion modes of Bi–O–Bi vibrations in distorted BiO_6_ octahedral units [37,38]. As a result of the combination of the corners of the TeO_4_, TeO_3+1_, and TeO_3_ units, the bands 494–512 cm^−1^ were aligned and matched with vibration connections Te–O–Te or O–Te–O [39,40,41]. The different IR peaks observed in the range of 500–800 cm^−1^ in the investigated glass may be related to the anti-symmetrical and symmetrical vibrations of TeO_2_ [41]. The peaks located in the range of 552–563 cm^−1^ were attributable to Bi–O^−^ bending vibration in BiO_6_ octahedral units [42]. The vibration of a continuous TeO_4_ trigonal bipyramid network was associated with bands detected in the 576–600 cm^−1^ range (tbp). These indicated the glass network’s more compacted connections [43]. The bands between 616 and 623 cm^−1^ were related to Ti–O bending vibrations [44]. The occurrence of Te–O_ax_ vibrations in the TeO_4_ tetrahedral units was linked to the development of the very strong band which occurred in the region of 648 to 654 cm^−1^. The vibrations of BO in TeO_3_/TeO_3+1_ units were responsible for the peaks between 671 and 674 cm^−1^ that were seen in all glass samples. The B–O–B connections in the borate network’s mode of vibration were responsible for the bands positioned in the range of 692–695 cm^−1^ [42]. The bands between 712 and 721 cm^−1^ were attributable to the NBO stretching modes present in TeO_3_ units [43]. The TeO_3_ trigonal pyramid (tp) and (TeO_3+1_) polyhedral units (Te_eq_–O)_as_ and (Te_eq_–O)_S_ vibrational modes were responsible for the bands in the 759–772 cm^−1^ range [42,43]. The bending vibrations of BO_4_ at 600 to 800 cm^−1^ and the B–O bond stretching vibrations of BO_4_ tetrahedral units are responsible for the bands that developed between 800 and 1200 cm^−1^.

The IR in the regions between 1200 and 1600 cm^−1^ were attributable to vibrations of B–O bonds from BO_3_ trigonal units [39]. Absorption from 912 to 925 cm^−1^ could be related to the stretching vibrations of B–O bond in BO_4_ units from diborate groups [44,45]. The peaks observed in the region of 990 to 1001 cm^−1^ may be attributable to the stretching vibrations of B–O–Bi linkages [37,38]. The IR peaks observed in the ranges of 1062–1067 cm^−1^ may be due to the stretching vibrations of B–O bonds in BO_4_ units from tri-, tetra-, and penta-borate groups [46]. The two IR peaks in the region of 1118 to 1120 cm^−1^ and 1162 to 1168 cm^−1^ in our investigated samples were attributable to TiO_4_ tetrahedral units [47]. Other IR peaks in the spectral ranges of 1241–1248 cm^−1^ were associated with the presence of asymmetrical stretching vibrations of B–O bonds in BO_3_ triangular units from pyro-borate groups [47]. The peaks which appeared in the range of 1280–1285 cm^−1^ were related to the B–O asymmetrical stretching vibration of (BO_3_)^−3^ units in meta- and ortho-borate groups [45]. The next absorption bands in the spectral ranges of 1348–1349 cm^−1^ were attributable to symmetrical stretching vibrations of B–O bonds in triangular BO_3_ units from meta-, pyro-, and ortho-borate groups [47,48]. The IR peak at 1375 cm^−1^ in all glass samples may have been due to the asymmetrical stretching vibrations of B–O bonds in triangular BO_3_ units [40]. The IR peaks in the ranges of 1401–1404 cm^−1^ can be attributed to the asymmetrical stretching vibrations of B–O triangles with BO_3_, B_2_O^−^, and stretching vibrations of borate triangles with NBO in various borate groups. The stretching vibrations of the B–O bonds in BO_3_ units obtained from different forms of borate groups were responsible for the bands seen between 1428 and 1430 cm^−1^ [40]. The peak at 1461 cm^−1^ may have been the result of three NBO oxygens in B–O–B links stretching in an anti-symmetrical manner [46]. All the IR bands in Table 4 were attributed to deconvolution FTIR spectra, as shown in Figure 5. Additionally, the ratio of TeO_4_ (tbp) to TeO_3_(tp) conversion was determined using the FTIR spectra. The ratio values of TeO_4_/TeO_3_ were 0.473, 0.504, 0.51, 0.52, and 0.53, corresponding to BBTTLa, BBTTCe, BBTTSm, BBTTEr, and BBTTYb, respectively. The ratio of transferring BO_4_ into BO_3_ was also determined from the deconvoluted FTIR spectra. Values of the ratio BO_4_/BO_3_ were 0.415, 0.5, 0.51, 0.52, and 0.53, which corresponded to BBTTLa, BBTTCe, BBTTSm, BBTTEr, and BBTTYb, respectively. The increases in the ratios of TeO_4_/TeO_3_ and BO_4_/BO_3_ show that the glass became more resistant as a result of the formation of additional bridging oxygens (BOs). Thus, the formation of BO sites with increased atomic numbers of rare earth elements resulted in a strictly dense glass structure that confirmed the increment in the ρ and decrement of V_m_ values for BBTTRE in the order similar to that La_2_O_3_ → Ce_2_O_3_ → Sm_2_O_3_ → Er_2_O_3_ → Yb_2_O_3_ → La_2_O_3_ glass samples, which was consistent with the parameter changes.

### 3.3. Attenuation Parameters

**Figure 6:**
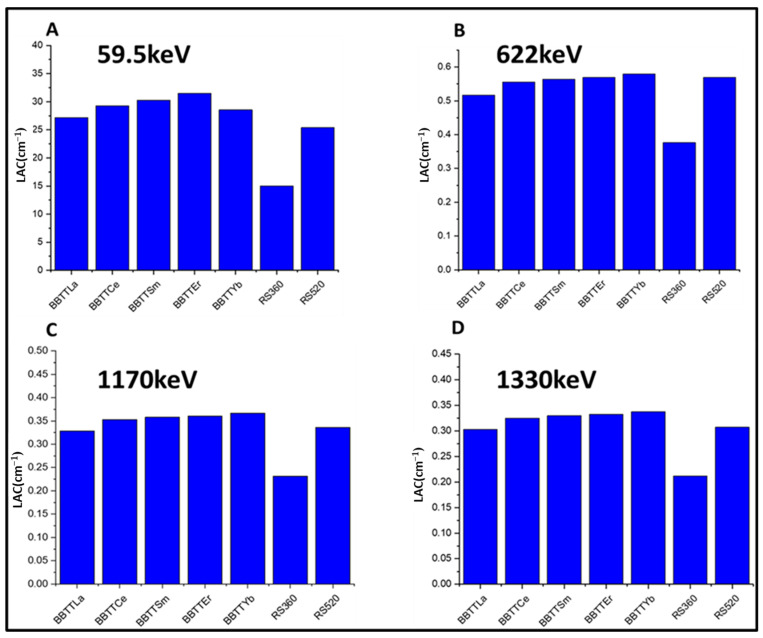
The calculated LAC for the glass samples compared with standard glass materials at energies: (**A**) 59.5; (**B**) 622; (**C**) 1170; and (**D**) 1330 keV.

**Figure 7:**
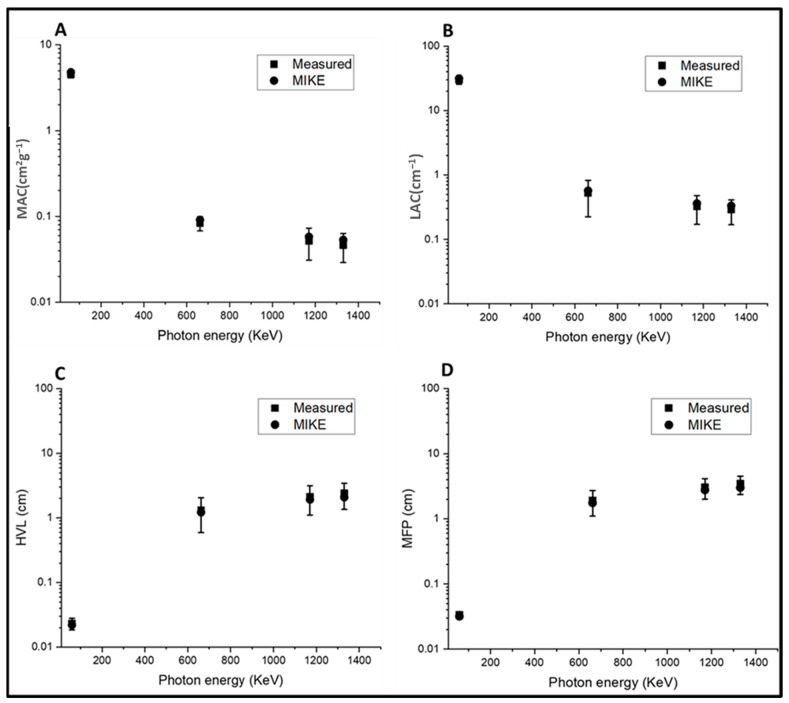
The measured and theoretical shielding parameters for the BBTTEr glass system at different photon energies (59.5, 622, 1170, and 1330 keV): (**A**) MAC; (**B**) LAC; (**C**) HVL; and (**D**) MFP.

The total mass attenuation coefficient, $\mu_{m}=\frac{\ln\frac{I_{o}}{I}}{\rho d}$, and the linear attenuation coefficient, $\mu =\frac{\ln\frac{I_{o}}{I}}{d}$, are calculated using the ratio between the intensities of the measured incident, I_0_, and the transmission radiation, I; d is the thickness of the shielding material. $\mathrm{HVL}=\frac{0.693}{\mu }$, and the MFP parameter was calculated as $\mathrm{MFP}=\frac{1}{\mu }$. Figure 6 shows the calculated linear attenuation coefficient (LAC) of the prepared glass for different energies (59.5, 622, 1170, and 1330 keV) compared with commercially available glass shielding materials, namely, RS360 and RS 520. For instance, the LAC value for BBTTYb glass exhibited the best shielding properties at the energies of 622, 1170, and 1330 keV at 59.5 keV, as compared with RS 360 and RS520 glass. Furthermore, the prepared glass (BBTTER) with a composition of 25B_2_O_3_ –20Bi_2_O_3_–45TeO_2_–7TiO_2_ (BBTT) modified by 3RE_2_O_3_ in mol%, where RE was La, Ce, Sm, Er, or Yb, was better than that reported in other glass systems modified with rare earth elements, such as 39B_2_O_3_–30PbO–20MO–10Bi_2_O_3_–1Eu_2_O_3_ (where M is K, Na, Ca, Sr, or Ba) [49], B_2_O_3_–CaO–TeO_2_–ZnO–ZnF_2_–Sm_2_O_3_ [50], and B_2_O_3_–SrCO_3_–Nb_2_O_3_–BaCO_3_–Dy_2_O_3_ [51]. Table 5 and Table 6 present the measured mass attenuation coefficients (MACs) of the prepared samples in comparison with the calculated theoretical values using the MIKE and WinXcom software. The HVL parameter signifies the material thickness that reduces the intensity of radiation by half. Herein, the values for HVL and MFP of the prepared glass were lower than that reported for commercial materials, such as window glass, serpentine, concrete, SCHOOT glass RS253, hematite serpentine, Ilmenite, and SCHOOT glass RS323 [52,53,54]. Figure 7 shows the measured values of MAC, LAC, HVL, and MFP for the BBTTEr glass at 59.5, 622, 1170, and 1330 keV, compared with the theoretical values calculated using MIKE software. The results showed good agreement between the measured mass attenuation coefficients and calculated values using MIKE software. Hence, the experimental attenuation results for the investigated prepared glass showed superior radiation shielding performance. Finally, we can estimate that the shielding parameters increased with the increasing ratios of TeO_4_/TeO_3_ and BO_4_/BO_3_ with bridging oxygens (BOs) of oxide glass, representing a candidate for the fabrication of superior shielding material.

## 4. Conclusions

Incorporating the rare earth ions La^+3^, Ce^+3^, Sm^+3^, Er^+3^, and Yb^+3^ as glass matrix modifiers, resulting in 25B_2_O_3_–20Bi_2_O_3_–45TeO_2_–7TiO_2_, it was found that the density of the studied glass increased from 5.67 to 6.31 gm.cm^−3^, n_b_ increased from 6.5 to 7.14 × 10^22^ m^−3^, and the OPD increased from 71.1 to 78.4 mol/L^−1^ with an increased atomic number of incorporated rare earth ions. This is due to the increased amount of bridging oxygen (BO) and decreased number of NBOs in the prepared glass, which also led to the increased E_opt_, from 1.71 to 2.8 eV, when La_2_O_3_ was replaced by Yb_2_O_3_. In addition, the molar polarizability, α_m_, decreased from 9.35 to 7.84 (Ă^3^), Λ decreased from 1.15 to 1.03, and the refractive index decreased from 2.69 to 2.45: this was due to the good agreement with the replacement of modifiers of La_2_O_3_ = 1.32 Ă^3^, Ce_2_O_3_ = 1.28 Ă^3^, Sm_2_O_3_ = 1.16 Ă^3^, Er_2_O_3_ = 0.89 Ă^3^, Yb_2_O_3_ = 0.86 Ă^3^, Λ_TeO4_^0^ = 0.99, Λ_TeO4_^−^ = 1.23, and Λ_TeO3_^−^ = 0.82, in the order of La^+3^ → Ce^+3^ → Sm^+3^ → Er^+3^ → Yb^+3^. The high refractive index, electronic polarizability, and optical basicity of the prepared glass containing La_2_O_3_ led to the achievement of significant third-order optical susceptibility. This glass may be used to produce high-quality optical nonlinear devices. The FTIR spectra confirmed the existence of TeO_4_, TeO_3_, BO_4_, BO_3_, BiO_6_, and TiO_4_ in the glass matrix. The glass containing Yb^3+^ ions had a high value of the TeO_4_ phase with BO. When compared with prepared glass, Yb^3+^-ion-containing glass exhibited higher MAC values and lower HVL values, which was directly related to its high shielding characteristics. This glass is an excellent choice for use in low-energy diagnostic applications as a transparent shielding material.

## Figures and Tables

**Table 1:** Compositions and codes of glass systems (45TeO_2_–25B_2_O_3_–20Bi_2_O_3_–7TiO_2_–3RE_2_O_3_) in mol%.

Sample Name	Glass Composition (mol%)									Sample Color
	TeO_2_	B_2_O_3_	Bi_2_O_3_	TiO_2_	La_2_O_3_	Ce_2_O_3_	Sm_2_O_3_	Er_2_O_3_	Yb_2_O_3_	
BBTTLa	45	25	20	7	3	―	―	―	―	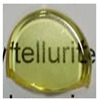
BBTTCe	45	25	20	7	―	3	―	―	―	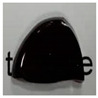
BBTTSm	45	25	20	7	―	―	3	―	―	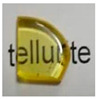
BBTTEr	45	25	20	7	―	―	―	3	―	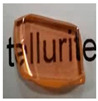
BBTTYb	45	25	20	7	―	―	―	―	3	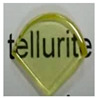

**Table 2:** Density (ρ), molar volume (V_m_), oxygen molar volume (V_O_), number of bonds (n_b_), average stretching force constant ($\bar{F}$), and oxygen packing density (OPD) of prepared glass samples.

Sample Name	ρ (g/cm^3^) ± 0.001	V_m_ (cm^3^/mol) ± 0.0056	Oxygen Molar Volume, V_o_ (cm^3^/mol) ± 0.2980	n_b_ × 10^22^ (m^−3^)	$\bar{F}$ (Nm^−1^)	OPD (mol/L) ± 0.078
BBTTLa	5.67	35	14.1	6.5	301.21	71.1
BBTTCe	6.083	32.5	13.1	7.05	300.2	76.2
BBTTSm	6.17	32.13	12.95	7.08	301.79	77.1
BBTTEr	6.21	32.11	12.94	7.09	301.48	77.2
BBTTYb	6.31	31.63	12.7	7.14	303.04	78.4

**Table 3:** Optical energy gap, E_opt_; refractive index, n; molar polarizability, α_m_; molar refraction, R_m_; oxide ion polarizability, $\alpha_{o}^{-2}$; metallization, M; and optical basicity, Λ, of the prepared glass.

Sample Name	E_opt_ (eV) ±0.01	n ±0.0001	R_m_ (cm^3^) ±0.0466	α_m,_ (Ă^3^) ±0.0182	$\alpha_{o}^{-2}$ (Ă^3^) ± 0.0065	M ± 0.0008	Λ ± 0.0025
BBTTLa	1.71	2.69	23.6	9.35	3.22	0.324	1.15
BBTTCe	2.47	2.61	21.5	8.52	2.88	0.339	1.09
BBTTSm	2.57	2.49	20.4	8.1	2.71	0.365	1.05
BBTTEr	2.68	2.46	20.2	8.008	2.68	0.371	1.04
BBTTYb	2.8	2.45	19.77	7.84	2.61	0.375	1.03

**Table 4:** The location of FTIR absorption bands corresponding to the structural bonds of the prepared glass samples.

Symbol	IR Bands Wavenumber (cm^−1^)	Assignments
a	370–400	Stretching mode of vibration of Bi–O–Bi linkages
b	430 440 458 425 430	Stretching vibration of La–O Stretching vibration of Ce–O Stretching vibration of Sm–O Stretching vibration of Er–O Stretching vibration of Yb–O
c	463–480	Bi–O–Bi vibration in distorted BiO_6_ octahedral units
b	494–512	Symmetrical stretching or bending vibrations of Te–O–Te or O–Te–O linkages
e	552–563	Bending vibration of Bi–O^−^ in BiO_6_ units
f	576–600	Vibration of the continuous network consisting of TeO_4_ tbp
g	616–623	Ti–O bending vibration
h	648–654	Symmetrical stretching vibration of Te–O_ax_ in TeO_4_ tetrahedral units
i	671–674	Stretching vibrations tellurium with BO of TeO_3_/TeO_3+1_ units
j	692–695	Bending vibrations of B–O–B linkages in the borate network
k	712–721	Stretching modes of NBO found on TeO_3_ and TeO_3+1_ units
l	759–772	Symmetrical and asymmetrical vibration of (Te_eq_–O) in TeO_3+1_ polyhedra or trigonal pyramid TeO_3_ (tp) units
m	912–925	Stretching vibrations of B–O bond in BO_4_ units from diborate groups
n	990–1001	Stretching vibrations of B–O–Bi linkages
o p	(1023–1028), (1062–1067)	Stretching vibrations of B–O bond in BO_4_ units from tri-, tetra- and penta-borate groups
q r	(1118–1120), (1162–1168)	TiO_4_
s t	(1247–1248), (1280–1285)	Asymmetric stretching vibrations of B–O bond in BO_3_ triangular units from meta-, pyro-, and ortho-borate groups
u v	(1317–1321), (1348–1349)	Symmetrical stretching vibrations of B–O bond in BO_3_ triangular units from meta-, pyro-, and ortho-borate groups
w	1375	Asymmetrical stretching vibrations of B–O bond in BO_3_ triangular units
x	1401–1404	Asymmetrical stretching vibrations of B–O triangle with BO_3_, B_2_O^−^ and stretching vibration of borate triangle with (NBO) in various borate groups
y	1428–1430	Stretching vibration of B–O bond in BO_3_ units from varied types of borate groups
z	1461	Anti-symmetric stretching vibrations with 3 NBO of B–O–B linkages

**Table 5:** The measured mass attenuation coefficients of BBTTLa, BBTTCe, and BBTTSm samples in comparison with the values calculated using MIKE software and theoretical estimated values (WinXcom).

Energy (keV)	Mass Attenuation Coefficient								
	BBTTLa			BBTTCe			BBTTSm		
	Exp	WinXCom	MIKE	Exp	WinXCom	MIKE	Exp	WinXCom	MIKE
59.5	4.528	4.717	4.7924	4.6070	4.741	4.8151	4.654	4.831	4.9056
662	0.084	0.090	0.0912	0.0839	0.091	0.0913	0.0841	0.0907	0.0914
1170	0.052	0.057	0.0580	0.0521	0.058	0.0580	0.0522	0.0579	0.0580
1330	0.046	0.053	0.0534	0.0463	0.053	0.0534	0.0464	0.0534	0.0535

**Table 6:** The measured mass attenuation coefficients of BBTTEr and BBTTYb samples in comparison with the values calculated using MIKE software and theoretical estimated values (WinXcom).

Energy (keV)	Mass Attenuation Coefficient					
	BBTTEr			BBTTYb		
	Exp	WinXCom	MIKE	Exp	WinXCom	MIKE
59.5	4.8245	4.988	5.0682	4.3090	4.4710	4.5282
662	0.0844	0.091	0.0917	0.0845	0.0912	0.0918
1170	0.0524	0.058	0.0581	0.0526	0.0580	0.0581
1330	0.0467	0.053	0.0535	0.0468	0.0535	0.0535

## Data Availability

Not applicable.
